# The association between family doctor contract services and the health of middle-aged and older people in China: an instrumental variables analysis

**DOI:** 10.1038/s41598-024-65621-0

**Published:** 2024-07-14

**Authors:** Weile Zhang, Min Su, Dongxu Li, Fan Yang, Zhengrong Li

**Affiliations:** School of Public Administration, Inner Mongolia University, Zhaojun Road, Yuquan District, Hohhot, 010070 Inner Mongolia China

**Keywords:** Health policy, Quality of life

## Abstract

Previous research on the association between Family Doctor Contract Services (FDCS) and health has only considered a single indicator of health and has not considered the endogeneity of independent variables. This study aimed to evaluate the association from a multidimensional perspective of the health of middle-aged and older people using the instrumental variables method and determine the underlying mechanisms. Using data from the 2018 China Health and Retirement Longitudinal Study surveys, a total of 19,438 sample was obtained. Health was measured by health related-quality of life (HR-QoL), subjective well-being, and cognitive function. The instrumental variables method was used to estimate the association. Mediation analysis was employed to analyze the underlying mechanisms. The results of the instrumental variables method showed a correlation between FDCS and health, such as HR-QoL (η = 33.714, *p* < 0.01), subjective well-being (η = 1.106, *p* < 0.05), and cognitive function (η = 4.133, *p* < 0.05). However, we found no evidence that FDCS improved physical health. We also identified reduced utilization of healthcare services and increased social activities as mediators of the effect of FDCS on health. The Chinese government should improve incentive-based initiatives to improve the quality of FDCS. Moreover, more attention needs to be paid to the multidimensional health of middle-aged and older people, especially vulnerable groups, such as older individuals and those in rural areas.

## Introduction

The family doctor system has been described by the World Health Organization (WHO) as the “most economical and appropriate” model of health care management recognized in most countries and regions. Family doctors, who provide primary and continuing care for all individuals, include physicians and other medical specialists in primary health care^1^. As the gatekeepers of residents’ health, family doctors play an important role in promoting hierarchical treatment^2^, enhancing the accessibility of health services^3^, and improving health^4^.

Existing practice and research show a positive association between family doctors and health. For example, in the United States, the family doctor system incorporated health management into community general practitioner services, which was effective in promoting the availability of health care^5^, reducing maternal mortality^6^, and informing related health policy and standard setting^7^. The Family Medicine Group (FMG) model of primary care in Quebec, Canada, revealed a significant relationship between family doctors and emergency department visits as well as hospital admissions^8^. Practice and research in Tehran also demonstrated that family doctors improved urban public health services^9^, and strikingly promoted health care access in rural areas^10^. Treharne et al. found when transgender people received supportive care from their family doctors in New Zealand, they experienced better mental health^11^.

China’s health care system reform in 2009 laid a good foundation for the implementation of the Family Doctor Contract Services (FDCS) system. Officially implemented in 2016, FDCS aimed to provide the public with a proactive, consistent, comprehensive, and affordable model of health accountability management in China^12^. At the end of 2021, approximately 1.435 million family doctors had been put in place (3.08 family doctors per 10,000 population), forming 431,000 teams to provide services to residents^13^. The FDCS services include basic medical care services, public health services, and individualized health management. According to the voluntarily signed contract and standardized service, family doctors establish a long-term service relationship with the families, enhancing the accessibility of contracted FDCS. By the end of 2020, the FDCS contracting rate for key populations increased from 28.33% in 2015 to 75.46%^14^. Moreover, most participants who contracted FDCS were willing to maintain contracts with their family doctors^15^. In addition, family doctors provide more than 95% of primary health care in rural China, indicting the services are easy to receive^16^. FDCS have made significant progress in promoting population health^17^. Li et al.found a significant and positive association between FDCS and HR-QOL among chronic patients in rural Shandong, China^18^. Xu et al. evaluated the effect of FDCS on the health management in Chinese type 2 diabetes mellitus patients, they found that patients participated in FDCS had a lower risk of diabetes-related complications than those who did not^19^. A cross-sectional survey in Shaanxi Province showed that the individuals with a contracted family doctor had significantly higher HR-QoL than those without^20^.

These studies highlight the importance of family doctors in improving population health. Nevertheless, current studies have some shortcomings. First, a single indicator or regional data is employed to measure population health. Second, current approaches to estimating the association between FDCS and health pay less attention to the endogeneity of family doctors, which may lead to bias. Third, an analysis of the mechanisms underlying the relationship between family doctors and health is lacking. Given the above limitations, this study aimed to: (1) based on national survey data, estimate the association between FDCS and health from a multidimensional perspective in China, (2) use the instrumental variables (IV) method to address the bias due to endogeneity, and (3) analyze the mechanisms underlying the association between FDCS and the health of middle-aged and older people using mediation analysis.

### Theoretical framework

**Figure 1:**
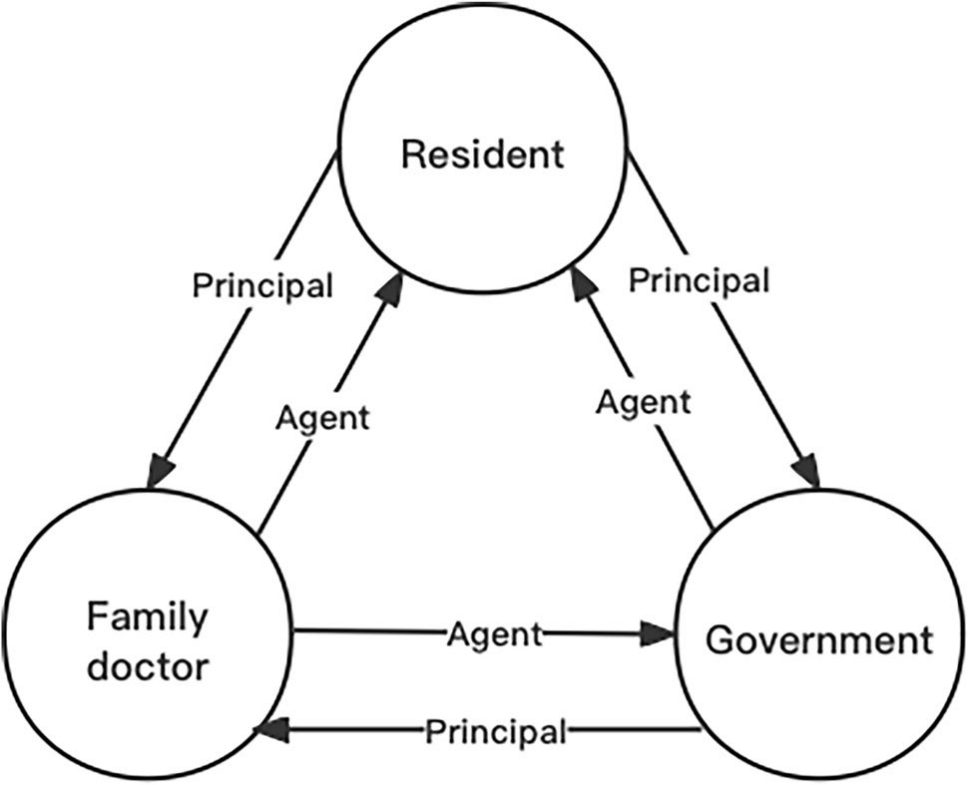
Theoretical framework. Author’s analysis.

The principal-agent theory is employed in the theoretical framework to explain the association between FDCS and the health of middle-aged and older people. The principal-agent theory has been widely used in the field of medicine to explain the healthcare payment contract^21^, medical choice^22^, and the doctor-patient relationship^23^. This paper applies the principal-agent theory and argues that there is a triple principal-agent relationship in FDCS, as shown in Fig. 1.

Solid arrows indicate the actual principal-agent relationship between residents, government, and family doctors. The first principal-agent relationship exists between residents and the government. As residents (the principal) were unable to employ family doctors directly, the government had the responsibility of protecting the public interest. Therefore, the government, as the agent, needed to formulate the FDCS that met the healthy needs of the residents, considering their willingness to contract, to ensure that they could receive more healthcare services from their family doctors.

The second principal-agent relationship exists between the government and family doctors. In China, the government could not provide health care directly, it entrusted family doctors in primary healthcare institutions to meet the needs of residents. The government promotes family doctors to provide services to residents through various means, such as incentives and supervision. In addition, family doctors play the role of the agent to provide health care services to residents directly. Moreover, FDCS currently covers basic medical services, basic public health services, and personalized services^24^.

The third principal-agent relationship exists between residents and family doctors. Residents act as principals and family doctors as agents. This relationship is characterized by a marked asymmetry of information, with family doctors having sophisticated medical expertise that the residents lack. In addition to information asymmetry, an anxiety asymmetry is also present. This means that when residents believe they might have a serious illness, they delegate their anxiety to their family doctor, providing an alternative explanation for residents seeking medical care^25^. Therefore, family doctors are obliged to use their skills competently to detect physical abnormalities among residents, alleviate their pain, and cure their illnesses.

## Methods

### Ethics approval and consent to participate

The China Health and Retirement Longitudinal Study (CHARLS) project was approved by the Peking University Biomedical Ethics Review Committee (IRB00001052-11015). Informed consent was obtained from all the study participants, and the data were anonymized for analysis. All methods were carried out in accordance with relevant guidelines and regulations.

### Data source

Data were obtained from the CHARLS, implemented by Peking University, which aimed to collect a nationally representative sample of middle-aged and older people to support aging and health-related research through a structured questionnaire (the data and questionnaire are available at http://charls.pku.edu.cn/). This study only included middle-aged and older people (aged ≥ 45 years) who participated in the survey in 2018. After removing the 228 samples younger than 45 years, a total of 19,438 valid samples were included.

## Measures

### Independent variable

In this study, the variable “Have you ever receive the paid family doctor services?” from the CHARLS was used to identify residents who had contracted FDCS.

### Dependent variables

The dependent variable was health, which includes HR-QoL, subjective well-being, and cognitive function^26–28^. To measure the HR-QoL, a new scale, the Short Form 36 (SF-36), was employed based on the CHARLS questionnaire. It is a 36-question comprehensive health survey that assesses eight health concepts used to define HR-QoL^29^. The scores of the eight subscales were summarized into a physical component score (PCS) and a mental component score (MCS), with higher PCS or MCS scores indicating higher levels of physical or mental health. Moreover, the variable “Please think about your life as a whole. How satisfied are you with it?” was used to identify the subjective well-being of the sample. Finally, the Mini Mental State Exam (MMSE), the most widely used neuropsychological scale for measuring cognitive function, was used^30^. In addition, there were missing values for dependent variables, such as HR-QoL (missing values = 1,789), PCS (missing values = 1,676), MCS (missing values = 1,548), subjective well-being (missing values = 1,546), and cognitive function (missing values = 9,997).

### Instrumental variable

“The combination of medical and healthcare service” was chosen as the instrumental variable. It was expressed in the CHARLS questionnaire as “Have you ever received the following home and community care services?”.

### Mediation variables

In addition, with reference to previous studies^31^, this study included the underlying mediation variables of healthcare service utilization (whether or not to use outpatient or inpatient services) and whether or not to participate in social activities.

### Control variables

The control variables included area, residence, gender, age, marital status, education, household income, insurance, smoke, drink, and chronic diseases. All Variables assignment see Table 1 for details.

**Table 1:** Variable assignment table.

Variables	Variables assignment
FDCS	NO = 0, YES = 1
HR-QoL (point)	Continuous variable
PCS (point)	Continuous variable
MCS (point)	Continuous variable
Subjective well-being (point)	Continuous variable
Cognitive function (point)	Continuous variable
The combination of medical and healthcare	NO = 0, YES = 1
Healthcare services utilization	
Outpatient	NO = 0, YES = 1
Inpatient	NO = 0, YES = 1
Social activities	NO = 0, YES = 1
Area	West = 0, Centre = 1, East = 2
Residence	Rural = 0, Urban = 1
Gender	Female = 0, Male = 1
Age (year)	Continuous variable
Marital status	Unmarried = 0, Married = 1
Education	Primary school or below = 0, Junior high school or above = 1
Household income (yuan)	Continuous variable
Insurance	NO = 0, YES = 1
Smoke	NO = 0, YES = 1
Drink	NO = 0, YES = 1
Chronic diseases	NO = 0, YES = 1

### Conceptual framework

**Figure 2:**
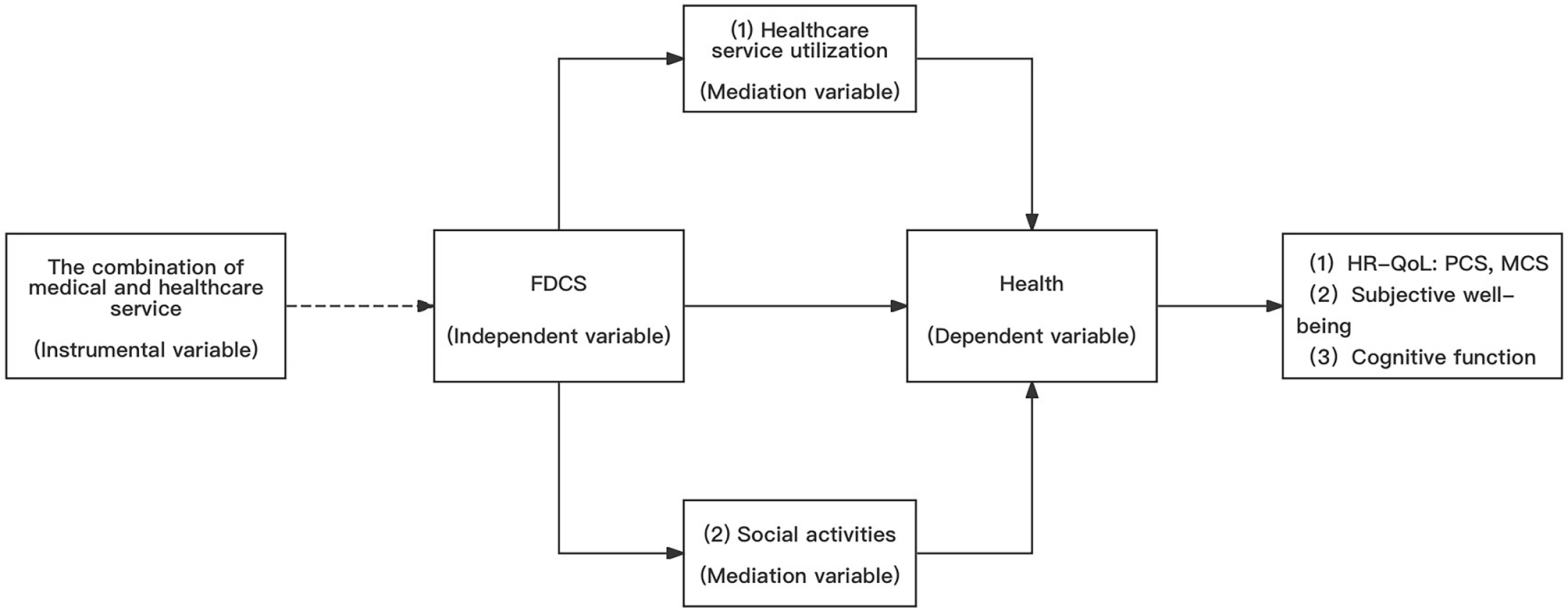
Conceptual framework. Source: Author’s analysis.

Figure 2 shows the conceptual framework of the study. To address the endogeneity of FDCS, we used “the combination of medical and healthcare service” as an instrumental variable to estimate the association of FDCS with health. In addition, the study hypothesizes that the FDCS affects the health of middle-aged and older people by influencing their “healthcare service utilization” and their “social activities”, which in turn affect their health (including HR-QoL, PCS, MCS, subjective well-being, and cognitive function).

### Model setting

This study determined the association of FDCS with health by constructing the following model:

1 $$Y_{it} = \alpha_{0} + \alpha_{1} FDCS_{it} + \alpha_{2} \beta_{it} + \sigma_{i} + \varepsilon_{it}$$

where $Y$ represents the explanatory variable of individual $i$ at the points $t$, representing the health of participants contracted FDCS; $FDCS$ represents the dummy variable of whether individual $i$ “received family doctor contract services” at time $t$, where 1 denotes received and 0 is not received; and ${\upbeta }_{i}$ represents the control variables, ${\upsigma }_{i}$ represents the area fixed effects, and ${\upvarepsilon }_{i}$ represents the random disturbance term.

### Instrumental variables method

When used as an explanatory variable, family doctors may cause endogeneity, leading to important information being overlooked when analyzing causality and thus to the overestimation or underestimation of the positive features of FDCS. In addition, population health may be affected by other factors, such as personal habits and the surrounding environment, which are unobservable.

In this study, the IV method was adopted to address endogeneity. It usually uses one or more instrumental variables that are related to the critical explanatory variable but not directly related to the outcome variable to identify the impacts of the exposure variable on the explained variable, which could lead to a consistent estimation. In this study, “the combination of medical and healthcare service” was chosen as instrumental variable. “The combination of medical and healthcare service” is provided by family doctors directly, but it not directly related to the health, so it meets the requirements of the IV method. The following regression model was set up for a two-stage least squares test:

2 $$Statues\_health\_\exp = \eta_{0} + \eta_{1} + yiyang_{h} + \delta X_{s,h} + \xi_{s,h}$$

3 $${Y}_{fd}={\uptheta }_{0}+{\uptheta }_{1}+ Statues\_health\_exp+\upbeta {X}_{s,h}+{\upmu }_{s,h}$$

Model (2) represents the first stage of the regression, with the dependent variable being health. $yiyang$ represents whether the participants received “the combination of medical and healthcare service.” Model (3) represents the results of the second-stage regression, where the dependent variable is family doctors, and $Statues\_health\_exp$ denotes estimates from the first-stage regression.

### Mediation mechanism analysis

Family doctors, through disease screening, may influence the utilization of health care services by middle-aged and older people, which in turn has an impact on their health. Second, through psychological counselling and health education, they may encourage residents to actively participate in social activities, which will expand their social network, prevent social isolation, and in turn affects the health of middle-aged and older people.

To further explore the mechanism of the relationship between FDCS and health, we conducted a mediation effect analysis using health care services utilization and social activities as mediation variables. According to the BK method^32^, we first tested the effect of FDCS on the health of middle-aged and older people; secondly, we tested the effect of FDCS on the mediation variables (i.e., Health care services utilization and social activities). Finally, we tested the effect of FDCS and the mediation variables on the health of middle-aged and older people. In this case, the test was stopped whenever one of the regression results was not significant, indicating that it could not pass the mediation mechanism test. Based on this, the following model is constructed:

4 $$Y_{it} = \alpha_{0} + \alpha_{1} FDCS_{it} + \alpha_{2} \beta_{it} + \sigma_{i} + \varepsilon_{it}$$

5 $${M}_{it}={\omega }_{0}+{\omega }_{1} {FDCS}_{it}+{\omega }_{2 }{\upbeta }_{it}+{\upsigma }_{i}+{\upvarepsilon }_{it}$$

6 $${Y}_{it}={\varphi }_{0}+{\varphi }_{1 }{FDCS}_{it}+{\varphi }_{\begin{array}{c}2\\ \end{array}}{ M}_{it}+{\varphi }_{2 }{\upbeta }_{it}+{\upsigma }_{i}+{\upvarepsilon }_{it}$$

where ${M}_{it}$ represents mediation variables of individual $i$ at the points $t$, representing health care services utilization and social activities of middle-aged and older people.

### Analytical strategy

All variables were presented as mean and standard deviation (S.D.). The ordinary least-squares (OLS) model was employed to estimate the impact of FDCS on the health of middle-aged and older people. All statistical analyses were performed using STATA statistical software version 15.1. A two-tailed *p*-value < 0.1 was considered statistically significant.

## Results

### Descriptive statistics of the sample

Table 2 shows the characteristics of the 19,438 samples. A total of 32.58% lived in the West and 40.15% lived in urban areas. 47.55% were male, and the average age was about 62 years. In addition, 34.44% of the participants had junior high school—level education or above, and 99.39% of the samples lived with their spouse. An overwhelming majority of participants were covered by insurance (97.03%), Nearly 5% of the participants smoked, and 34% drunk. It was reported that 49.29% participants had chronic diseases, 16.47% and 16.88% participants utilized the outpatient services and inpatient services, respectively, and 53.01% participants had social activities. See Table 2 for details.

**Table 2:** Basic characteristics of the sample.

Variables	Total (n = 19,438)	Contracted (n = 800)	No contracted (n = 18,638)	*P/t*
Area				< 0.001
West	6333 (32.58)	281 (35.12)	6052 (32.47)	
Centre	6535 (33.62)	216(27.00)	6319 (33.90)	
East	6570 (33.80)	303 (37.88)	6267 (33.62)	
Residence				0.102
Urban	7805 (40.15)	299 (37.38)	7506 (40.27)	
Rural	11633 (59.85)	501 (62.62)	11132 (59.73)	
Gender				0.002
Male	9242 (47.55)	424 (52.00)	8818 (47.31)	
Female	10196 (52.45)	376 (47.00)	9820 (52.69)	
Age (years)				− 3.784
Mean ± S.D	61.99 ± 10.03	63.31 ± 10.15	61.94 ± 10.02	
Education				0.009
Junior high school and above	6695 (34.44)	310 (38.75)	6385 (34.26)	
Elementary school and below	12743 (65.56)	490 (61.25)	12253 (65.74)	
Marital status				0.691
Marriage	19320 (99.39)	796 (99.50)	18524 (99.39)	
Unmarried	118 (0.61)	4 (0.50)	114 (0.61)	
Insurance				0.012
Yes	18860 (97.03)	788 (98.50)	18072 (96.96)	
No	578 (2.97)	12 (1.50)	566 (3.04)	
Ln (house income) (yuan)				− 0.548
Mean ± S.D	8.87 ± 2.60	8.92 ± 2.50	8.87 ± 2.61	
Smoke				0.611
Yes	898 (4.62)	34 (4.25)	864 (4.64)	
No	18540 (95.38)	766 (95.75)	17774 (95.36)	
Drink				0.925
Yes	6566 (33.78)	269 (33.62)	6297 (33.79)	
No	12872 (66.22)	531 (66.38)	12341 (66.21)	
Chronic diseases				< 0.001
Yes	9581 (49.29)	465 (58.13)	9116 (48.91)	
No	9857 (50.71)	335 (41.88)	9522 (51.09)	
Outpatient				< 0.001
Yes	3201 (16.47)	181 (22.62)	3020 (16.20)	
No	16237 (83.53)	619 (77.38)	15618 (83.80)	
Inpatient				0.001
Yes	3282 (16.88)	170 (21.25)	3112 (16.70)	
No	16156 (83.12)	630 (78.75)	15526 (83.30)	
Social activities				0.037
Yes	10305 (53.01)	453 (56.62)	9852 (52.86)	
No	9133 (46.99)	347 (43.38)	8786 (47.14)	

(1) Pearson’s chi-squared test was used for categorical variables and t-test was used for continuous variables.

(2) Due to the high number of zeros and extreme values of the ‘household income’ variable, it was treated logarithmically, with ‘Ln’ denoting the logarithm with ‘e’ as the base.

## Regression results

Table 3 shows the estimated results of the association of FDCS with the health. Participants who received the FDCS had an increase in HR-QoL of 1.084 points (*p* < 0.05). FDCS significantly increased mental health by 2.196 points (*p* < 0.01). In addition, participants who received the FDCS had improved subjective well-being and cognitive functioning by 0.087 points (*p* < 0.01) and 0.354 points (*p* < 0.01), respectively. See Table 3 for details.

**Table 3:** Regression results of the association of FDCS with the health.

Variables	(1)	(2)	(3)	(4)	(5)
	HR-QoL	PCS	MCS	Subjective well-being	Cognitive function
FDCS	1.084**	− 0.023	2.196***	0.087***	0.354***
(No = 0)	(0.509)	(0.671)	(0.603)	(0.031)	(0.106)
Gender	3.244***	5.033***	1.427***	0.024*	0.280***
(Female = 0)	(0.227)	(0.295)	(0.279)	(0.014)	(0.052)
Resident	− 1.423	− 0.931	− 0.694	0.273	1.522**
(Rural = 0)	(3.436)	(4.430)	(4.769)	(0.178)	(0.679)
Age	− 0.226***	− 0.234***	− 0.238***	0.006***	− 0.030***
(Continuous variables)	(0.012)	(0.015)	(0.014)	(0.001)	(0.003)
Education	2.880***	1.982***	3.817***	− 0.033**	0.620***
(Elementary school and below = 0)	(0.233)	(0.305)	(0.280)	(0.014)	(0.050)
Marriage	3.135*	3.527*	2.745	0.043	0.538
(Without spouse)	(1.672)	(1.977)	(1.949)	(0.096)	(0.440)
Insurance	− 0.540	− 2.133***	1.037	0.150***	0.148
(No = 0)	(0.627)	(0.789)	(0.760)	(0.044)	(0.171)
Chronic	− 4.809***	− 7.651***	− 2.059***	− 0.122***	0.031
(No = 0)	(0.197)	(0.254)	(0.241)	(0.012)	(0.044)
Smoke	− 0.202	0.311	-0.811	− 0.092***	− 0.059
(No = 0)	(0.495)	(0.647)	(0.586)	(0.029)	(0.105)
Drink	2.136***	1.760***	2.559***	0.017	− 0.006
(No = 0)	(0.229)	(0.299)	(0.280)	(0.014)	(0.051)
Ln (household income)	0.952***	0.865***	1.037***	0.025***	0.066***
	(0.045)	(0.058)	(0.055)	(0.003)	(0.011)
Constant term	70.545***	76.720***	63.919***	2.583***	7.475***
	(4.377)	(5.663)	(5.708)	(0.237)	(0.928)
Area fixed effects	YES	YES	YES	YES	YES
Community fixed effects	YES	YES	YES	YES	YES
*N*	17,649	17,762	17,890	17,892	9,441
R-squared	0.268	0.217	0.207	0.082	0.157

(1) The value in parentheses is the standard error.

(2) *, **, and *** represent statistical significance at the 10%, 5%, and 1% levels, respectively.

(3) Due to the presence of missing values for the dependent variable, the regression analysis was analyzed again using multiple interpolation to supplement the sample size. The results in Table S1 shows that the direction of the coefficients of the dependent variable are consistent with the above results and results remain significant, indicating the results are robust.

### Endogeneity analysis

Table 4 shows the results of the effects of FDCS on the health using the IV method. The F-values for the IV in the first-stage regression results were greater than 10, rejecting the initial hypothesis that the IV was weakly instrumental and demonstrating the validity of the IV. The results showed a correlation between FDCS and the health, including HR-QoL (η = 33.714, *p* < 0.01), subjective well-being (η = 1.106, *p* < 0.05), and cognitive function (η = 4.133, *p* < 0.05). Compared to the results of the OLS regression, FDCS improved HR-QoL, subjective well-being, and cognitive function by 32.63, 1.019, and 3.779 points, respectively, indicating that endogeneity in the model caused the OLS regression to underestimate the association between FDCS and the health. See Table 4 for details.

**Table 4:** Results of the endogeneity analysis.

Variables	HR-QoL		Subjective well-being		Cognitive function	
	Model (2)	Model (3)	Model (2)	Model (3)	Model (2)	Model (3)
FDCS		33.714***		1.106**		4.133**
Instrumental variable	0.047***		0.047***		0.053***	
Control variables	YES	YES	YES	YES	YES	YES
Area fixed effects	YES	YES	YES	YES	YES	YES
Constant term	0.034	70.157***	0.041***	2.564***	− 0.055	7.807***
*N*	17,649	17,649	17,892	17,892	9,441	9,441
Endogeneity test	0.000		0.000		0.000	
Weak instrumental variable test	48.253		49.243		28.342	
R-squared	0.094		0.092		0.109	

(1) Instrumental variable refers to ‘the combination of medical and healthcare’.

(2) *, **, and *** represent statistical significance at the 10, 5, and 1% levels, respectively.

### Analysis of heterogeneity

To further analyze the impact of FDCS on health in detail, we analyzed heterogeneity in terms of age, place of residence, and the presence of chronic diseases. Specifically, FDCS significantly improved the health for those 45 ~ 65 years. Regarding differences in place of residence, FDCS improved the health of rural residents more than that of urban residents overall. Regarding the presence of chronic disease, FDCS improved health improved the health of residents with chronic diseases than counterparts. See Table 5 for details.

**Table 5:** Results of the heterogeneity analysis.

Variables	Age difference		Place of residence difference		presence of chronic diseases	
	45 ~ 65 years old	≥ 65 years old	Rural	Urban	No	Yes
HR-QoL	1.135* (0.650)	0.309 (0.804)	0.850 (0.640)	1.436* (0.836)	0.410 (0.815)	1.452** (0.675)
PCS	− 0.183 (0.862)	− 0.422 (1.069)	− 0.498 (0.839)	0.735 (1.114)	− 1.220 (1.102)	0.489 (0.874)
MCS	2.462*** (0.787)	1.060 (0.939)	2.123*** (0.783)	2.295** (0.941)	2.170** (0.972)	2.389*** (0.794)
Subjective well-being	0.113*** (0.042)	0.042 (0.047)	0.108** (0.040)	0.057 (0.056)	0.039 (0.047)	0.117*** (0.043)
Cognitive function	0.203 (0.138)	0.500*** (0.181)	0.526*** (0.145)	0.129 (0.155)	0.161 (0.165)	0.527*** (0.144)

*, **, and *** represent statistical significance at the 10, 5, and 1% levels, respectively.

### Underlying mechanisms

S2 demonstrates the effect of FDCS on the mediation variables. S3 presents the effect of mediation variables on the health. See appendix for details. Table 6 shows the underlying mechanisms between FDCS and the health of middle-aged and older people. In terms of HR-QoL, the coefficient of FDCS increased after the inclusion of the mediation variable of healthcare service utilization (*p* < 0.05). This indicates that FDCS improved the health by reducing their healthcare service utilization. Regarding subjective well-being, the coefficient and significance level of FDCS increased after the inclusion of the mediation variables of healthcare service utilization (*p* < 0.01) and social activities (*p* < 0.05). This implies that FDCS improved health by reducing healthcare service utilization and increasing social activities. In addition, with respect to cognitive function, the coefficient for FDCS increased when the social activities variable was added (*p* < 0.01). This indicated that FDCS increased the cognitive function by guiding residents to increase social activities. See Table 6 for details.

**Table 6:** Results of the test of the underlying mechanism.

Variables	HR-QoL				MCS			Subjective well-being				Cognitive function
FDCS	1.203**	1.294**	0.226		2.266***	2.257***	0.624	0.092***		0.090***	0.085**	0.350***
(No = 0)	(0.505)	(0.575)	(0. 465)		(0.604)	(0.603)	(0.472)	(0.031)		(0.031)	(0.013)	(0.106)
Outpatient		− 3.988***			− 1.322***			− 0.102***				
(No = 0)		(0.291)			(0.330)			(0.017)				
Inpatient	− 4.868***					− 2.460***				− 0.114***		
(No = 0)	(0.276)					(0.340)				(0.017)		
Social activities			11.314***				21.072***				0.029***	0.094***
(No = 0)			(0.187)				(0.114)				(0.013)	(0.048)
Area fixed effects	YES	YES	YES		YES	YES	YES	YES		YES	YES	YES
Community fixed effects	YES	YES	YES		YES	YES	YES	YES		YES	YES	YES
*N*	17,649	17,649	17,649		17,890	17,890	17,890	17,892		17,892	17,892	9,441
R-squared	0.282	0.277	0.398		0.207	0.209	0.528	0.084		0.084	0.082	0.157

*, **, and *** represent statistical significance at the 10, 5, and 1% levels, respectively.

## Discussion

In this study, we found a positive association between FDCS and the health of middle-aged and older people in China. We used IV method to estimate and test the association, which may help to provide a robust and convincing result. Moreover, heterogeneity in the effect of FDCS was present according to age, residence, and presence of chronic diseases. Finally, we conducted a detailed mediation analysis and demonstrated that healthcare service utilization and social activities were important mediating factors.

Principal-agent theory can provide a possible explanation for the result that FDCS significantly improved most health outcomes. FDCS had a significant effect on HR-QoL. The results were similar to those found in the USA, Japan, and rural China^18,33,34^. In terms of the government, recently health system reforms tend to encourage initial contact with primary healthcare providers in China^35^, which may substantially affect health. Family doctors are incentivized by government regulation and market accountability in addition to government incentives^36^. As a result, motivated family doctors are committed to providing high-quality medical services, thus further improving the health. However, similar to another study^17^, we found no evidence that FDCS led to improvements in physical health. The possible explanation is that the coverage of welfare programs designed by the government only includes government employees rather than family doctors^37^. Therefore, family doctors with lower salary levels and unequal promotion systems^38^ often experience burnout^39^, resulting in low motivation to improve the physical health of middle-aged and older people. Acknowledged information asymmetry and thinking frameworks^40^ also influence family doctors’ decision-making on the physical health. In addition, one study showed that MCS is more related to HR-QoL than PCS^41^, and residents with poorer health are more motivated to participate in FDCS^42^. However, the older the person, the worse the health, the more time and effort family doctors need to improve PCS. The FDCS was implemented after 2016, so residents’ PCS may not be significantly improved in the short period of 2 years.

This study found a significant association between FDCS and subjective well-being. This can be explained by the fact that the government provides family doctors with comprehensive training to improve their skills, competence, and confidence, resulting in reduced dissatisfaction with local medical services among middle-aged and older people and alleviated healthcare inequality^43^, which in turn improves resident’ subjective well-being. Family doctors could provide better services to the population and maintain independence^44^. For example, they provide specialized treatment, increasing the continuity of care and reducing admission rates^45^, thus increasing their subjective well-being.

The results demonstrated that FDCS had a positive effect on the cognitive function of the population. However, previous studies concluded that family doctors are not effective in improving cognitive function due to low levels of knowledge and skills regarding dementia^46^. A possible explanation for this discrepancy is that the government trains family doctors in dementia care and requires them to provide dementia screening for the population^47^, which contributes to the early detection and prevention of cognitive impairment. In addition, family doctors showed generally positive attitudes towards people with dementia and put their dementia training into practice, educating and correcting residents’ and their families’ misconceptions about the disease to improve cognitive function.

The results were heterogeneous in terms of age, residence, and presence of chronic diseases. First, the impact of FDCS on health was greater for the population aged < 65 years than for individuals aged 45 ~ 65 years. Health was inversely related to age. Moreover, older people are prone to multiple chronic diseases, and patients with multiple chronic diseases and their family doctors have a higher rate of therapeutic inertia^48^, which has a negative impact on the health of older people. Second, the impact of FDCS on health was greater for the rural residents than for the urban residents. One possible explanation is that rural areas are often associated with a lack of sufficient and high-quality medical resources and qualified primary care providers^49^. Therefore, the effects of FDCS are more pronounced for the rural population than for the urban population. Third, FDCS had a greater positive impact on the health of patients with chronic diseases than those without. Family doctors provide targeted health management measures for patients with chronic diseases, such as the provision of chronic disease service packages, health knowledge promotion, and monitoring of health indicators to improve population health.

The mediation results showed that FDCS improved health by influencing healthcare service utilization and social activates. This can be explained by the fact that family doctors able to identify and address health risks in primary healthcare, reducing healthcare service utilization^50^and further improving health expectations of residents. In addition, FDCS can improve health through influencing resident’ social activities. The results support some previous studies investigating the relationship between various aspects of social relationships and health^51,52^. Family doctors provide a variety of health interventions, such as exercise guidance and psychological guidance, which could encourage residents to participate in social activities, increase exposure to an interactive environment, facilitate the early detection of cognitive decline, and improve their health.

This study also has several limitations. First, all the data were collected using a self-reporting approach, which may introduce recall bias. In addition, the availability of measured perspectives on population health was limited by the pre-specified questions in the survey, and we did not control for some potential unobserved confounding factors. Finally, this was a cross-sectional study, hence, the data may not be comprehensive enough to identify changes in the association.

## Conclusion

FDCS played a positive role in improving the health of middle-aged and older people in China. The government should improve incentive-based initiatives to improve the quality of services and pay more attention to the health of middle-aged and older people. Moreover, more attention needs to be directed toward vulnerable groups, such as older individuals and those in rural China.

### Supplementary Information

Supplementary Information.

## Data Availability

This data was derived from the CHARLS. They are opened to everyone. Researchers who want to use these data can visit http://charls.pku.edu.cn/. We had added questionnaires content in the appendix.
