# Supplementary material for: The association between family doctor contract services and the health of middle-aged and older people in China: an instrumental variables analysis
Source: Sci Rep. 2024 Jul 14;14:16229. doi: 10.1038/s41598-024-65621-0 (PMC11247085; doi:10.1038/s41598-024-65621-0)
Supplement: Supplementary file 1 — Supplementary Information. [file 41598_2024_65621_MOESM1_ESM.docx]

S1 Regression results of the association of FDCS with the health after multiple interpolation

| Variables | (1) | (2) | (3) | (4) | (5) |
| --- | --- | --- | --- | --- | --- |
|  | HR-QoL | PCS | MCS | Subjective well-being | Cognitive function |
| FDCS | 0.993^**^ | −0.066 | 2.051^***^ | 0.081^***^ | 0.292^**^ |
| (No = 0) | (0.502) | (0.663) | (0.605) | (0.031) | (0.114) |
| Gender | 3.222^***^ | 5.029^***^ | 1.416^***^ | 0.024^*^ | 0.226^***^ |
| (Female = 0) | (0.227) | (0.295) | (0.275) | (0.014) | (0.054) |
| Resident | 0.692 | −0.090 | 1.295 | 0.259 | 0.284 |
| (Rural = 0) | (3.342) | (4.272) | (4.647) | (0.187) | (0.686) |
| Age | −0.240^***^ | −0.238^***^ | −0.243^***^ | 0.006^***^ | −0.029^***^ |
| (Continuous variables) | (0.011) | (0.015) | (0.013) | (0.001) | (0.003) |
| Education | 2.918^***^ | 1.999^***^ | 3.837^***^ | −0.033^**^ | 0.673^***^ |
| (Elementary school and below = 0) | (0.229) | (0.300) | (0.280) | (0.014) | (0.046) |
| Marriage | 3.555^**^ | 3.471^*^ | 3.639^**^ | 0.028 | 0.470 |
| (Without spouse) | (1.519) | (1.875) | (1.752) | (0.091) | (0.353) |
| Insurance | −0.474 | −2.071^***^ | 1.123 | 0.144^***^ | 0.249 |
| (No = 0) | (0.621) | (0.803) | (0.738) | (0.043) | (0.196) |
| Chronic | −5.050^***^ | −7.830^***^ | −2.271^***^ | −0.125^***^ | 0.037 |
| (No = 0) | (0.195) | (0.249) | (0.240) | (0.012) | (0.044) |
| Smoke | −0.399 | 0.198 | −0.995^*^ | −0.097^***^ | −0.039 |
| (No = 0) | (0.481) | (0.645) | (0.574) | (0.029) | (0.090) |
| Drink | 2.228^***^ | 1.745^***^ | 2.710^***^ | 0.018 | 0.025 |
| (No = 0) | (0.231) | (0.301) | (0.278) | (0.014) | (0.049) |
| Ln (household income) | 0.964^***^ | 0.881^***^ | 1.048^***^ | 0.025^***^ | 0.081^***^ |
|  | (0.045) | (0.059) | (0.054) | (0.003) | (0.010) |
| Constant term | 68.451^***^ | 75.681^***^ | 61.221^***^ | 2.618^***^ | 8.654^***^ |
|  | (4.123) | (5.323) | (5.473) | (0.241) | (0.874) |
| Area fixed effects | YES | YES | YES | YES | YES |
| Community fixed effects | YES | YES | YES | YES | YES |
| *N* | 19,438 | 19,438 | 19,438 | 19,438 | 19,438 |

Note: (1) The value in parentheses is the standard error; (2) *, **, and *** represent statistical significance at the 10, 5, and 1% levels, respectively.

S2 Results of the effect of FDCS on mediation variables

| **Variables** | Utilization of outpatient services | Utilization of inpatient services | Social activities |
| --- | --- | --- | --- |
| FDCS | 0.224*** | 0.115** | 0.201*** |
|  | (0.055) | (0.056) | (0.050) |
| Control variables | YES | YES | YES |
| Area fixed effects | YES | YES | YES |
| Community fixed effects | YES | YES | YES |
| *N* | 19,438 | 19,438 | 19,438 |
| R-squared | 0.075 | 0.114 | 0.101 |

Note: (1) The value in parentheses is the standard error; (2) *, **, and *** represent statistical significance at the 10, 5, and 1% levels, respectively.

S3 Results of the impact of mediation variables on the health of middle-aged and older people

| **Variables** | HR-QoL | | | MCS | | | Subjective well-being | | | Cognitive function | | |
| --- | --- | --- | --- | --- | --- | --- | --- | --- | --- | --- | --- | --- |
| Outpatient | −3.969*** |  |  | −1.288** |  |  | −0.100*** |  |  | 0.047 |  |  |
| (No = 0) | (0.258) |  |  | (0.330) |  |  | (0.017) |  |  | (0.060) |  |  |
| Inpatient |  | −4.859*** |  |  | −2.444*** |  |  | −0.113*** |  |  | −0.052 |  |
| (No = 0) |  | (0.276) |  |  | (0.341) |  |  | (0.017) |  |  | (0.064) |  |
| Social activities |  |  | 11.317*** |  |  | 21.079*** |  |  | 0.030*** |  |  | 0.097*** |
| (No = 0) |  |  | (0.187) |  |  | (0.195) |  |  | (0.013) |  |  | (0.048) |
| Area fixed effects | YES | YES | YES | YES | YES | YES | YES | YES | YES | YES | YES | YES |
| Community fixed effects | YES | YES | YES | YES | YES | YES | YES | YES | YES | YES | YES | YES |
| *N* | 17,649 | 17,649 | 17,649 | 17,890 | 17,890 | 17,890 | 17,892 | 17,892 | 17,892 | 9,441 | 9,441 | 9,441 |
| R-squared | 0.277 | 0.281 | 0.398 | 0.207 | 0.209 | 0.527 | 0.083 | 0.084 | 0.082 | 0.156 | 0.156 | 0.156 |

Note: (1) The value in parentheses is the standard error; (2) *, **, and *** represent statistical significance at the 10, 5, and 1% levels, respectively.
